# The maternal microbiome during pregnancy and allergic disease in the offspring

**DOI:** 10.1007/s00281-017-0652-y

**Published:** 2017-10-16

**Authors:** Peter J Vuillermin, Laurence Macia, Ralph Nanan, Mimi LK Tang, Fiona Collier, Susanne Brix

**Affiliations:** 1 Deakin University, Geelong, Australia; 2 Barwon Health, Geelong, Australia; 3 Murdoch Childrens Research Institute, Parkville, Australia; 4 Centre for Food and Allergy Research, Parkville, Australia; 5 Charles Perkins Centre, Discipline of Pathology, School of Medical Sciences, The University of Sydney, Sydney, New South Wales Australia; 6 The Royal Children’s Hospital, Melbourne, Parkville, Australia; 7 Department of Biotechnology and Biomedicine, Technical University of Denmark, Kongens Lyngby, Denmark

**Keywords:** Maternal, Microbiome, In utero, Immune programming, Allergy, Asthma

## Abstract

There is substantial epidemiological and mechanistic evidence that the increase in allergic disease and asthma in many parts of the world in part relates to changes in microbial exposures and diet acting via the composition and metabolic products of the intestinal microbiome. The majority of research in this field has focused on the gut microbiome during infancy, but it is increasingly clear that the maternal microbiome during pregnancy also has a key role in preventing an allergy-prone immune phenotype in the offspring. The mechanisms by which the maternal microbiome influences the developing fetal immune system include alignment between the maternal and infant regulatory immune status and transplacental passage of microbial metabolites and IgG. Interplay between microbial stimulatory factors such as lipopolysaccharides and regulatory factors such as short-chain fatty acids may also influence on fetal immune development. However, our understanding of these pathways is at an early stage and further mechanistic studies are needed. There are also no data from human studies relating the composition and metabolic activity of the maternal microbiome during pregnancy to the offspring’s immune status at birth and risk of allergic disease. Improved knowledge of these pathways may inform novel strategies for tackling the increase in allergic disorders in the modern world.

## Introduction

The co-evolution of animals and their commensal microorganisms has resulted in a symbiosis in which the enteric microbiome provides essential stimuli for the maturation and function of the immune system [1]. Accordingly, there is an intense interest in the relationship between taxonomic depletion and altered metabolic activity of the gut microbiome [2–4] and the concordant increase in immune-related disease in Western societies [2–5]. It is well recognised that the infant’s microbiome during early postnatal life plays a central role in healthy immune development, but there is also mounting evidence that the maternal microbiome during pregnancy has a profound impact on fetal immune development and in turn the infant’s predisposition to allergic disease and asthma. It is a teleologically appealing proposition to consider the mechanisms that may exist to prepare the infant for the microbial and antigenic environment that will be encountered during and following birth. Does the mother ready the developing infant for the specific environment they will be born into and are the changes in the maternal gut microbiome during pregnancy [6] relevant to healthy fetal immune development? The aim of this review is to consider current thinking and evidence regarding the mechanisms by which the maternal gut microbiome during pregnancy influences fetal immune development and the potential relevance of these pathways to the prevention of allergic disease.

## Epidemiological evidence linking the maternal microbiome and allergic disease in the offspring

There is substantial evidence that exposure to a farming environment during early life is associated with diverse bacterial experience and reduced risk of allergic sensitisation and asthma [7]. In the PASTURE study, it was shown that living in a farming environment was associated with both increased environmental endotoxin and reduced asthma incidence [8]. Building on this, the PARSIFAL [9] and GABRIELA [10] studies confirmed that children living on farms had a lower prevalence of asthma and other atopic disease in comparison to children from suburban areas, and that this association related to increased exposure to environmental microorganisms in farming homes [11]. Further analysis of these studies indicated that the effect on asthma was largely explained by exposure to cows, straw, consumption of raw cow’s milk [12] and exposure to livestock [13].

Although it seems likely that contemporaneous exposure to a microbially rich environment prevents the expression of allergic disease in children [14], maternal exposures during pregnancy are also likely to be relevant. In PARSIFAL, maternal exposure to stables during pregnancy was clearly associated with decreased allergic sensitisation amongst the offspring, whereas current exposure had much weaker effects [15]. Consistent with this, a large cross-sectional study found that the protective effect of prenatal livestock exposure on risk of asthma and hay fever symptoms remained unchanged following adjustment for postnatal exposure, whereas the protective effect of livestock exposure during the first 2 years of postnatal life was substantially attenuated by controlling for prenatal exposure [16]. Of note though, these and other related epidemiological studies are limited by high levels of correlation between pre- and postnatal microbial exposure, making it difficult to tease apart the importance of the two periods. Moreover, environmental exposures provide only a proxy for the maternal and infant microbiome, and there are currently no published human studies which enable independent evaluation of the importance of prenatal maternal and postnatal infant microbiomes to the prevention of allergic disease.

A range of studies have established clear links between differences in innate and adaptive immunity at birth and subsequent allergic disease and asthma, which suggests that in utero immune development is likely to be relevant to the prevention of allergy and asthma. In the 1990s, several studies reported links between decreased IFNg (Th1) responses in cord blood mononuclear cells and subsequent allergic disease [17–19]. Subsequent studies have established links between increased innate immune responsiveness [20, 21], deficits in regulatory T cell number, function and responsiveness [21–23] and subsequent allergic outcomes. While there is insufficient evidence to show that the association between microbial exposure during pregnancy and decreased allergic disease in the offspring is mediated by these immune parameters in the newborn, it is clear that infants of mothers exposed to a farming environment display different innate immune gene expressions [24] as well as increased number and efficiency of regulatory T cells [25].

## Mechanisms by which the maternal microbiome during pregnancy may influence fetal immune development

### Alignment between maternal and infant immunity

Alignment between maternal and infant immunity may be one mechanism linking the maternal microbiome to the offspring’s risk of allergic disease. There is a substantial body of evidence supporting transplacental immune regulation during pregnancy. Maternal IgGs loaded with e.g. microbial components from the mother cross the fetal–maternal barrier by an active process from 13 weeks gestation [26], conveying temporary passive immunity [27] and influencing fetal innate immune development [28]. In contrast, cellular components are generally separated by the placenta, with some leakage in both directions without preference toward a specific cell type [29]. This cellular leakage is functionally important, as maternal cells residing in fetal lymph nodes induce fetal regulatory T cells that suppress antimaternal immunity [30]. Transplacental immune regulation may be further mediated by cytokines, hormones [31], through bacterial products such as short-chain fatty acids [32] or lipopolysaccharides (LPS) [33, 34].

Santner-Nanan et al. have demonstrated a strong correlation of peripheral blood T_reg_ cells between the mother and the fetus [35]. In contrast, there was no significant T_reg_ cell correlation between the father and the fetus, implicating that the specific context of pregnancy, i.e. the placental environment, rather than haploidentical genetic parental similarity to the fetus, is responsible for this correlation. Maternal infant alignment in T_reg_ cells appeared to be mediated by IL-10, a pleiotropic cytokine with potent immunoregulatory properties [36]. T_reg_ cells are characterised by increased expression of the IL-10 receptor-α (IL-10RA), making them more sensitive to the effects of IL-10. The IL-10 regulates Bcl-2 expression in T_reg_ cells, which could contribute to T_reg_ cell survival in both the mother and the infant [35].

In the context of alignment between maternal and infant T_reg_, it is relevant to consider emerging evidence linking pregnancies complicated by preeclampsia with increased risk of asthma and allergic sensitisation in the offspring [37]. Impaired growth of the fetal thymus precedes the onset of clinically detectable preeclampsia [38]; and preeclampsia has been associated with decreased peripheral blood T_reg_ in both the mother [39] and newborn [40]. One potential shared antecedent is the maternal microbiome and its metabolic products, including short-chain fatty acids (SCFAs).

### Production of SCFAs in the maternal gut

Over recent years, there has been an intense interest in the relationship between diet, the composition and metabolic products of the intestinal microbiome and immune-related disease [41]. In particular, it has been clearly demonstrated that bacteria in the large intestine ferment dietary fibre to produce SCFAs, which in turn have a profound influence on a range of inflammatory diseases [42, 43], T_reg_ biology [44–46], dendritic cell (DC) biology [42, 47] and/or epithelial integrity [48, 49]. To date, however, only one study has directly investigated the relationship between maternally derived SCFAs and fetal immune programming. Thorburn et al. found that pregnant mice fed on high-fibre diet or treated with the SCFA acetate in drinking water during pregnancy had offsprings protected from allergic airways disease [32]. The beneficial effects of fibre and acetate were mediated in utero as supplementation during lactation had no effect. These beneficial effects were mediated through epigenetic effects with acetate increasing H4 acetylation in the FoxP3 promoter region, which correlated with increased T_reg_ numbers in both mothers and offsprings. High-fibre-fed and acetate-treated pregnant mice had fetuses with differential gene expression in the lungs compared to control mice. There are currently no equivalent data from human studies.

### Maternal IgG and fetal innate immune development

Maternal IgG may play a key role in mediating the association between the maternal microbiome and fetal immune development. Of the five immunoglobulin classes, maternal IgG is the only antibody that significantly crosses the human placenta [26, 50]. The active transport of IgG occurs via the neonatal Fc receptor (FcRn) within the syncytiotrophoblast (ST) cells at the surface of the chorionic villi of the placenta. Once bound to the FcRn receptor, IgG is packaged in endosomes and protected from degradation until it dissociates into the fetal circulation [50, 51].

This materno-fetal IgG transport is an important mechanism that confers passive humoral immunity to the fetus, so that after birth, the infant is protected against infections while its own immune system develops [26, 50]. Allergen-specific maternal IgG also plays a role in the induction of immune tolerance in infant [52]. Until recently, maternal IgG transfer during gestation had only been linked to fetal humoral immunity, but there is now good evidence that maternal IgG also plays a crucial in fetal innate immune development [28]. Aguero et al. colonised pregnant germ-free mice with a genetically engineered *Escherichia coli* with a limited lifespan, such that the mothers were germ free again by the time of birth. Colonisation with *E*. *coli* during pregnancy increased the numbers of innate leukocytes (NKp46 + innate lymphoid cells (ILC)) within the offspring Peyer’s patches during the postnatal period. At the same time, maternal carriage of *E*. *coli* was associated with attenuated inflammatory responses (TNF-α and IL-6) to stimulation with LPS, which may be relevant to the prevention of the hyperresponsive innate phenotype associated with allergic disease in humans [20, 21]. Similar effects in the offspring could be produced if germ-free dams were infused with serum from *E*. *coli* colonised mothers, but not if the serum was depleted of IgG. Further to this, maternal antibodies enhanced the retention and transmission of targeted microbial molecules produced by the *E*. *coli*, and these molecules were responsible for the increase in offspring innate leukocytes [28].

### Early exposure to lipopolysaccharide (endotoxin) producing bacteria

The maternal intestinal microbiome may also confer protection against allergic disease and asthma in the offspring via TLR-dependent pathways [34]. Interactions between bacterially derived LPS (endotoxin) and TLR4 are of particular interest given consistent evidence that associations between microbial exposures and allergic outcomes in humans are modified by polymorphisms in the TLR4 gene [53–56]. However, the relationship between LPS exposure and allergic diseases is complex, with some epidemiological studies finding that environments with high levels of endotoxin are associated with decreased allergic disease [14, 57], while others link endotoxin exposure to increased allergic disease [58]. This may relate to diversity amongst LPS molecules in regard to their TLR4 stimulatory capacity [59, 60]. Several different endotoxin isoforms have been identified that vary in the extent of acylation of the TLR4-binding lipid A portion of the molecule. While the penta-acylated lipid A variant produced by many bacteria confers a degree of TLR4 inhibition, the hexa- or hepta-acylated lipid A variants are potent stimulators of TLR4 signalling, resulting in promotion of T helper type 1 (Th1) responses [61, 62]. Moreover, immune cells may mount T_reg_, Th2 or Th17 responses following exposure to stimulatory LPS, depending on the presence of various other immunoregulatory ligands [63]. Bacterial isolates from farming environments (cow sheds) have high levels of stimulatory (hexa- or hepta-acylated lipid A variants) LPS variants [64]. Increased T_reg_ numbers and suppressive functions in offspring of farming mothers [25] may reflect an interaction between Th1 driving stimulators and immunoregulatory mediators. More specifically, it is an intriguing possibility that the maternal microbiome may provide immunostimulatory components such as hexa-acylated LPS, in combination with immunoregulatory microbial metabolites such as SCFAs, to promote T_reg_ development and immune tolerance in the developing fetus.

## Manipulating the maternal microbiome during pregnancy to prevent allergic disease and asthma

Given the increasing evidence that infant gut microbial composition plays a pivotal role in the development of immune responses, probiotic and/or prebiotic supplementation during pregnancy and infancy has been investigated as an approach to modify early gut colonisation and prevent allergic diseases. Probiotics are defined as ‘live microorganisms that, when administered in adequate amounts, confer a health benefit on the host’. The most widely used probiotic bacteria are from the genus *Lactobacillus* and *Bifidobacteria*. Prebiotics are defined as ‘a selectively fermented ingredient that allows specific changes, both in the composition and/or activity in the gastrointestinal microbiota that confers benefits upon host well being and health’. The term synbiotic refers to combination of probiotics and prebiotics.

Recently, high-quality meta-analyses have concluded that probiotic supplementation, when administered during both the prenatal and postnatal period, is effective for prevention of eczema, IgE associated eczema, atopic sensitisation and food sensitisation, particularly in infants with a family history of allergic disease [65–67]. There has however been just one study evaluating probiotic supplementation (with *Lactobacillus rhamnosus* GG) during the prenatal period alone which showed no beneficial effects for prevention of eczema, IgE associated eczema, atopic sensitisation or food sensitisation [68]. Meta-analyses of probiotic administration solely to infants during the postnatal period have yielded conflicting results with some finding no beneficial preventive effects on eczema [69] or increased risk of atopic sensitisation [66]. These findings suggest that a prenatal component of treatment is important for beneficial effects, highlighting the important influence of maternal factors in infant immune programming. Most probiotic prevention studies have involved infants at increased risk of allergic disease due to presence of allergic disease in a first-degree relative, so it remains uncertain whether probiotic supplementation will be effective in infants without a family history of allergic disease. The use of probiotic mixtures appears to be more effective than single species of either *Lactobacillus* or *Bifidobacteria* [65], and infants delivered by caesarean section may receive greater benefit from prenatal/postnatal probiotic supplementation that infants delivered vaginally [66].

Evidence from animal studies clearly shows that maternal supplementation with prebiotics during pregnancy may reduce the features of allergic disease in the offspring. For example, in mice, maternal consumption of non-digestible oligosaccharides during pregnancy is associated with decreased dermatitis [70] and allergic airways disease [32, 71] and in pigs, as increased Th1 and T_reg_ immunity [72]. However, while there are a number of related trials in progress, the efficacy of prebiotic supplementation during pregnancy in humans remains uncertain.

Meta-analyses have found no consistent beneficial effects from probiotic supplementation (either prenatal, postnatal or both) for prevention of asthma, rhinoconjunctivitis or food allergy [65, 67]. Thus, despite promising findings from some individual studies, it is difficult to recommend routine use of probiotics for allergy prevention [73]. It is however important to recognise that probiotic effects are species-specific; consequently, pooling of studies that use different probiotic species in meta-analyses has been criticised [74]. Indeed, even strains within one species may have very different biological properties [75]. Only a small proportion of potentially suitable microbes have been evaluated. Almost all current probiotics are Gram-positive, with the exception of the *E*. *coli* strain Nissle [34, 76]. Our understanding of the impact of probiotics on the overall composition and metabolic activity of the gut microbiome is very limited. There is much to be learnt about the multitude of organisms yet to be evaluated as potential probiotics, including Gram-negative and anaerobic bacteria and parasites, alone and in combination, as well the importance of diet-microbiome interactions [77]. This gap in knowledge provides an exciting opportunity for new studies to develop novel and sophisticated strategies for restoring a healthy microbiome.

## Conclusions

A range of epidemiological and mechanistic evidence supports the contention that the composition and metabolic products of the maternal microbiome play a key role in programming tolerogenic immune phenotypes in the offspring at birth in turn decreasing the risk of allergic disease and asthma. The mechanisms involved may include alignment between maternal and infant immunity as well as transplacental passage maternal IgG and microbial metabolites. There is also an evolving understanding of the importance of LPS variants and their interaction with the developing immune system, and the metabolite and cytokine milieu in which these interactions occur. Interplay between a ‘Westernised’ microbiome and the modern diet may be crucial. At present, however, there is insufficient evidence to support the implementation of interventions to manipulate the maternal microbiome during pregnancy for prevention of allergic disease and or asthma in the offspring. Given dramatic advances in our ability to characterise the composition and metabolic activity of the microbiome, studies are required that link direct measures of the maternal microbiome in humans, with immune phenotypes in the offspring and subsequent allergic outcomes. There has been a vast amount of work done on the infant microbiome, but the maternal microbiome during pregnancy remains relatively underinvestigated, particularly in humans, and may be of fundamental importance to the prevention of allergic disease and asthma in the modern environment.

## References

[1] Hooper LV, Littman DR, Macpherson AJ (2012). Interactions between the microbiota and the immune system. Science.

[2] De Filippo C, Cavalieri D, Di Paola M (2010). Impact of diet in shaping gut microbiota revealed by a comparative study in children from Europe and rural Africa. Proc Natl Acad Sci U S A.

[3] Schnorr SL, Candela M, Rampelli S (2014). Gut microbiome of the Hadza hunter-gatherers. Nat Commun.

[4] Sonnenburg ED, Smits SA, Tikhonov M (2016). Diet-induced extinctions in the gut microbiota compound over generations. Nature.

[5] Bach J-F (2002). The effect of infections on susceptibility to autoimmune and allergic diseases. N Engl J Med.

[6] Koren O, Goodrich JK, Cullender TC (2012). Host remodeling of the gut microbiome and metabolic changes during pregnancy. Cell.

[7] Genuneit J (2012). Exposure to farming environments in childhood and asthma and wheeze in rural populations: a systematic review with meta-analysis. Pediatr. Allergy Immunol.

[8] Mutius V, Braun-Fahrländer S (2000). Exposure to endotoxin or other bacterial components might protect against the development of atopy. Clin Exp Allergy.

[9] Alfvén T, Braun-Fahrländer C, Brunekreef B (2006). Allergic diseases and atopic sensitization in children related to farming and anthroposophic lifestyle—the PARSIFAL study. Allergy.

[10] Genuneit J, Büchele G, Waser M (2011). The GABRIEL Advanced Surveys: study design, participation and evaluation of bias. Paediatr Perinat Epidemiol.

[11] Ege MJ, Mayer M, Normand A-C (2011). Exposure to environmental microorganisms and childhood asthma. N Engl J Med.

[12] Wlasiuk G, Vercelli D (2012). The farm effect, or: when, what and how a farming environment protects from asthma and allergic disease. Curr Opin Allergy Clin Immunol.

[13] Brunekreef B, Von Mutius E, Wong GK (2012). Early life exposure to farm animals and symptoms of asthma, rhinoconjunctivitis and eczema: an ISAAC Phase Three Study. Int J Epidemiol.

[14] Stein MM, Hrusch CL, Gozdz J (2016). Innate immunity and asthma risk in Amish and Hutterite farm children. N Engl J Med.

[15] Ege MJ, Bieli C, Frei R (2006). Prenatal farm exposure is related to the expression of receptors of the innate immunity and to atopic sensitization in school-age children. J Allergy Clin Immunol.

[16] Douwes J, Cheng S, Travier N (2008). Farm exposure in utero may protect against asthma, hay fever and eczema. Eur Respir J.

[17] Tang MLK, Kemp AS, Hill DJ, Thorburn J (1994). Reduced interferon-γ secretion in neonates and subsequent atopy. Lancet.

[18] Prescott SL, Macaubas C, Holt BJ (1998). Transplacental priming of the human immune system to environmental allergens: universal skewing of initial T cell responses toward the Th2 cytokine profile. J Immunol.

[19] Macaubas C, de Klerk NH, Holt BJ (2003). Association between antenatal cytokine production and the development of atopy and asthma at age 6 years. Lancet.

[20] Tulic MK, Hodder M, Forsberg A (2011). Differences in innate immune function between allergic and nonallergic children: new insights into immune ontogeny. J Allergy Clin Immunol.

[21] Zhang Y, Collier F, Naselli G (2016). Cord blood monocyte-derived inflammatory cytokines suppress IL-2 and induce nonclassic “T(H)2-type” immunity associated with development of food allergy. Sci Transl Med.

[22] Smith M, Tourigny MR, Noakes P (2008). Children with egg allergy have evidence of reduced neonatal CD4(+)CD25(+)CD127(lo/−) regulatory T cell function. J Allergy Clin Immunol.

[23] Ismail IH, Boyle RJ, Mah L-J (2014). Reduced neonatal regulatory T cell response to microbial stimuli associates with subsequent eczema in high-risk infants. Pediatr Allergy Immunol.

[24] Roduit C, Wohlgensinger J, Frei R (2011). Prenatal animal contact and gene expression of innate immunity receptors at birth are associated with atopic dermatitis. J Allergy Clin Immunol.

[25] Schaub B, Liu J, Höppler S (2009). Maternal farm exposure modulates neonatal immune mechanisms through regulatory T cells. J Allergy Clin Immunol.

[26] Palmeira P, Quinello C, Silveira-Lessa AL (2012). IgG placental transfer in healthy and pathological pregnancies. Clin Dev Immunol.

[27] Firan M, Bawdon R, Radu C (2001). The MHC class I-related receptor, FcRn, plays an essential role in the maternofetal transfer of gamma-globulin in humans. Int Immunol.

[28] Gomez de Agüero M, Ganal-Vonarburg SC, Fuhrer T (2016). The maternal microbiota drives early postnatal innate immune development. Science.

[29] Loubière LS, Lambert NC, Flinn LJ (2006). Maternal microchimerism in healthy adults in lymphocytes, monocyte/macrophages and NK cells. Lab Investig.

[30] Mold JE, Michaëlsson J, Burt TD (2008). Maternal alloantigens promote the development of tolerogenic fetal regulatory T cells in utero. Science.

[31] PrabhuDas M, Bonney E, Caron K (2015). Immune mechanisms at the maternal-fetal interface: perspectives and challenges. Nat Immunol.

[32] Thorburn AN, McKenzie CI, Shen S (2015). Evidence that asthma is a developmental origin disease influenced by maternal diet and bacterial metabolites. Nat Commun.

[33] Voltolini C, Battersby S, Etherington SL (2012). A novel antiinflammatory role for the short-chain fatty acids in human labor. Endocrinology.

[34] Conrad ML, Ferstl R, Teich R (2009). Maternal TLR signaling is required for prenatal asthma protection by the nonpathogenic microbe Acinetobacter lwoffii F78. J Exp Med.

[35] Santner-Nanan B, Straubinger K, Hsu P (2013). Fetal-maternal alignment of regulatory T cells correlates with IL-10 and Bcl-2 upregulation in pregnancy. J Immunol.

[36] Mosser DM, Zhang X (2008). Interleukin-10: new perspectives on an old cytokine. Immunol Rev.

[37] Stokholm J, Sevelsted A, Anderson UD, Bisgaard H (2017). Preeclampsia associates with asthma, allergy, and eczema in childhood. Am J Respir Crit Care Med.

[38] Eviston DP, Quinton AE, Benzie RJ (2012). Impaired fetal thymic growth precedes clinical preeclampsia: a case-control study. J Reprod Immunol.

[39] Santner-Nanan B, Peek MJ, Khanam R (2009). Systemic increase in the ratio between Foxp3+ and IL-17-producing CD4+ T cells in healthy pregnancy but not in preeclampsia. J Immunol.

[40] Vargas-Rojas MI, Solleiro-Villavicencio H, Soto-Vega E (2016). Th1, Th2, Th17 and Treg levels in umbilical cord blood in preeclampsia. J Matern Fetal Neonatal Med.

[41] Tan JK, McKenzie C, Mariño E (2017). Metabolite-sensing G-protein coupled receptors—facilitators of diet-related immune regulation. Annu Rev Immunol.

[42] Trompette A, Gollwitzer ES, Yadava K (2014). Gut microbiota metabolism of dietary fiber influences allergic airway disease and hematopoiesis. Nat Med.

[43] Maslowski KM, Vieira AT, Ng A (2009). Regulation of inflammatory responses by gut microbiota and chemoattractant receptor GPR43. Nature.

[44] Smith PM, Howitt MR, Panikov N et al (2013) The microbial metabolites, short-chain fatty acids, regulate colonic Treg cell homeostasis. Science. 10.1126/science.1237947 10.1126/science.1241165 PMC3807819 23828891

[45] Arpaia N, Campbell C, Fan X (2013). Metabolites produced by commensal bacteria promote peripheral regulatory T-cell generation. Nature.

[46] Furusawa Y, Obata Y, Fukuda S (2013). Commensal microbe-derived butyrate induces the differentiation of colonic regulatory T cells. Nature.

[47] Tan J, McKenzie C, Vuillermin PJ (2016). Dietary fiber and bacterial SCFA enhance oral tolerance and protect against food allergy through diverse cellular pathways. Cell Rep.

[48] Fukuda S, Toh H, Hase K (2011). Bifidobacteria can protect from enteropathogenic infection through production of acetate. Nature.

[49] Macia L, Tan J, Vieira AT (2015). Metabolite-sensing receptors GPR43 and GPR109A facilitate dietary fibre-induced gut homeostasis through regulation of the inflammasome. Nat Commun.

[50] Kumpel BM, Manoussaka MS (2012). Placental immunology and maternal alloimmune responses. Vox Sang.

[51] Schneider H, Miller RK (2010). Receptor-mediated uptake and transport of macromolecules in the human placenta. Int J Dev Biol.

[52] Polte T, Hennig C, Hansen G (2008). Allergy prevention starts before conception: maternofetal transfer of tolerance protects against the development of asthma. J Allergy Clin Immunol.

[53] Custovic A, Marinho S, Simpson A (2012). Gene–environment interactions in the development of asthma and atopy. Expert Rev Respir Med.

[54] Böttcher MF, Hmani-Aifa M, Lindström A (2004). A TLR4 polymorphism is associated with asthma and reduced lipopolysaccharide-induced interleukin-12 (p70) responses in Swedish children. J Allergy Clin Immunol.

[55] Werner M, Topp R, Wimmer K (2003). TLR4 gene variants modify endotoxin effects on asthma. J Allergy Clin Immunol.

[56] Seo J, Kim H, Kang M (2012). Gene-environment interaction between early life exposure and CD14, TLR4, IL13 in development of allergic diseases or Atopy. J Allergy Clin Immunol.

[57] Braun-Fahrländer C, Riedler J, Herz U (2002). Environmental exposure to endotoxin and its relation to asthma in school-age children. N Engl J Med.

[58] Thorne PS, Kulhánková K, Yin M (2005). Endotoxin exposure is a risk factor for asthma: the national survey of endotoxin in United States housing. Am J Respir Crit Care Med.

[59] Brix S, Eriksen C, Larsen JM, Bisgaard H (2015). Metagenomic heterogeneity explains dual immune effects of endotoxins. J Allergy Clin Immunol.

[60] Vatanen T, Kostic AD, d’Hennezel E (2016). Variation in microbiome LPS immunogenicity contributes to autoimmunity in humans. Cell.

[61] Larsen JM, Steen-Jensen DB, Laursen JM (2012). Divergent pro-inflammatory profile of human dendritic cells in response to commensal and pathogenic bacteria associated with the airway microbiota. PLoS One.

[62] Park BS, Song DH, Kim HM (2009). The structural basis of lipopolysaccharide recognition by the TLR4-MD-2 complex. Nature.

[63] Millien VO, Lu W, Shaw J (2013). Cleavage of fibrinogen by proteinases elicits allergic responses through toll-like receptor 4. Science.

[64] Debarry J, Garn H, Hanuszkiewicz A (2007). Acinetobacter lwoffii and Lactococcus lactis strains isolated from farm cowsheds possess strong allergy-protective properties. J Allergy Clin Immunol.

[65] Zuccotti G, Meneghin F, Aceti A (2015). Probiotics for prevention of atopic diseases in infants: systematic review and meta-analysis. Allergy.

[66] Zhang G-Q, Hu H-J, Liu C-Y (2016). Probiotics for prevention of atopy and food hypersensitivity in early childhood: a PRISMA-compliant systematic review and meta-analysis of randomized controlled trials. Medicine.

[67] Cuello-Garcia CA, Brożek JL, Fiocchi A (2015). Probiotics for the prevention of allergy: a systematic review and meta-analysis of randomized controlled trials. J Allergy Clin Immunol.

[68] Boyle RJ, Ismail IH, Kivivuori S (2011). Lactobacillus GG treatment during pregnancy for the prevention of eczema: a randomized controlled trial. Allergy.

[69] Tang MLK, Lahtinen SJ, Boyle RJ (2010). Probiotics and prebiotics: clinical effects in allergic disease. Curr Opin Pediatr.

[70] Fujiwara R, Takemura N, Watanabe J, Sonoyama K (2010). Maternal consumption of fructo-oligosaccharide diminishes the severity of skin inflammation in offspring of NC/Nga mice. Br J Nutr.

[71] Hogenkamp A, Knippels LMJ, Garssen J, van Esch BCAM (2015). Supplementation of mice with specific nondigestible oligosaccharides during pregnancy or lactation leads to diminished sensitization and allergy in the female offspring. J Nutr.

[72] Le Bourgot C, Ferret-Bernard S, Le Normand L (2014). Maternal short-chain fructooligosaccharide supplementation influences intestinal immune system maturation in piglets. PLoS One.

[73] Campbell D, Vale S, Smith J (2016). ASCIA-P5: ASCIA guidelines for infant feeding and allergy prevention. Intern Med J.

[74] Szajewska H, Shamir R, Turck D (2015). Recommendations on probiotics in allergy prevention should not be based on pooling data from different strains. J Allergy Clin Immunol.

[75] Ley RE (2016). Gut microbiota in 2015: Prevotella in the gut: choose carefully. Nat Rev Gastroenterol Hepatol.

[76] Adam E, Delbrassinne L, Bouillot C (2010). Probiotic *Escherichia coli* Nissle 1917 activates DC and prevents house dust mite allergy through a TLR4-dependent pathway. Eur J Immunol.

[77] Kovatcheva-Datchary P, Nilsson A, Akrami R (2015). Dietary fiber-induced improvement in glucose metabolism is associated with increased abundance of Prevotella. Cell Metab.

